# What Is a Mild Winter? Regional Differences in Within-Species Responses to Climate Change

**DOI:** 10.1371/journal.pone.0132178

**Published:** 2015-07-09

**Authors:** Sebastian G. Vetter, Thomas Ruf, Claudia Bieber, Walter Arnold

**Affiliations:** Department of Integrative Biology and Evolution, Research Institute of Wildlife Ecology, University of Veterinary Medicine, Vienna, Savoyenstraße 1, Vienna, Austria; University of Sassari, ITALY

## Abstract

Climate change is known to affect ecosystems globally, but our knowledge of its impact on large and widespread mammals, and possibly population-specific responses is still sparse. We investigated large-scale and long-term effects of climate change on local population dynamics using the wild boar (*Sus scrofa* L.) as a model species. Our results show that population increases across Europe are strongly associated with increasingly mild winters, yet with region-specific threshold temperatures for the onset of exponential growth. Additionally, we found that abundant availability of critical food resources, e.g. beech nuts, can outweigh the negative effects of cold winters on population growth of wild boar. Availability of beech nuts is highly variable and highest in years of beech mast which increased in frequency since 1980, according to our data. We conclude that climate change drives population growth of wild boar directly by relaxing the negative effect of cold winters on survival and reproduction, and indirectly by increasing food availability. However, region-specific responses need to be considered in order to fully understand a species’ demographic response to climate change.

## Introduction

Despite an increasing understanding of the impact of global climate change on a broad range of ecosystems [1] there is a lack of long-term and large-scale biological data-sets necessary to gain a more complete understanding of how ecosystems are affected by climate change [2]. Additionally, most studies dealing with the impact of climate change on animals are distributional studies [3], showing that many species respond to increasing ambient temperatures with range shifts [2, 4]. Some studies, however, also found that local populations of non-migratory species of birds and fishes differ in their thermal tolerance and their response to temperature changes [5, 6]. Such differences between different populations might be due to physiological differences caused by local adaptation or phenotypic plasticity. In fact, it is known that body mass is affected by the general climatic conditions with individuals living in cold climates being larger [7]. Some studies on bird species even reported changes in body mass in response to climate change [8, 9]. However, very little is known on how large mammals with a wide distribution range respond to climate change and whether those responses differ between populations [10, 11]. Thus, it is unclear whether results of studies on the impact of climate on single populations can be generalized throughout a species’ distribution range.

The wild boar is an excellent model to investigate this topic. First, it is one of the most widely distributed mammals in the world [12, 13], with recent significant growth documented for many populations [14, 15]. As wild boars occupy a broad range of habitat types and climatic zones with a number of genetically distinct ecotypes [16, 17], there is a high potential for region-specific responses to climate change in this species. Second, the wild boar has an enormous reproductive capacity, and thus the potential for remarkable population growth when environmental conditions become more favourable, e.g. [18]. Given an average litter size in Europe of ~ 5 juveniles [19], reproductive output in the wild boar is much larger than in other ungulates of similar size [20]. Furthermore, if food availability is high, female boar can reach sexual maturity yet in their year of birth [21], and maximum life-span is high, i.e. up to 12 years in natural habitats [12]. Third, climatic conditions are known to affect reproduction [21], as well as survival [22] of wild boar.

Climate change, however, could affect wild boar populations not only directly through changes in temperature and precipitation, but also indirectly by affecting important biotic factors such as food availability. Such ecological impacts of climate change at the community level have received less attention than direct responses to abiotic changes but are important for a holistic understanding of how climate change affects both, species and communities [2]. Although an omnivorous species, the wild boar strongly relies on tree seeds like beech nuts, *Fagus sylvatica* L., or acorns, *Quercus spec*. L. in large parts of its distribution range in Europe [23]. Fructification in these trees is highly pulsed (seed masting), but the frequency of masting events has significantly increased over the past decades in parts of Europe (e.g. Austria, see below), probably as a consequence of climate change [24].

**Fig 1:**
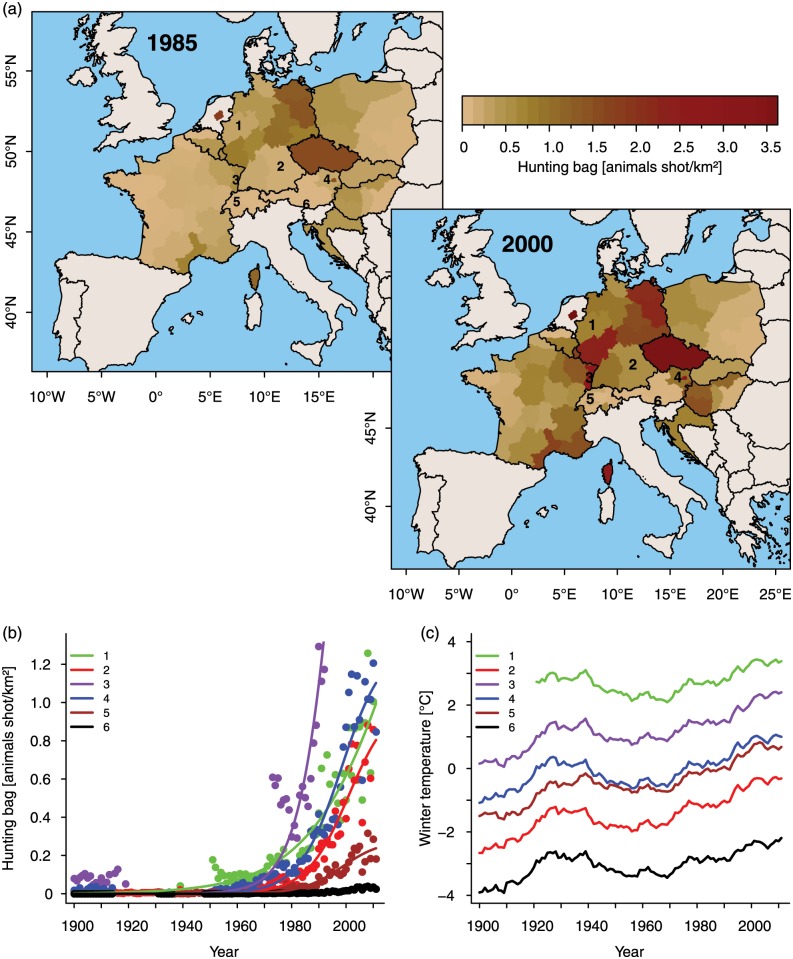
Increase of European wild boar populations and mean winter temperatures. (a) Color-coded wild boar densities in various regions 1985 and 2005, respectively. For six exemplary regions time courses of wild boar hunting bags (b) as well as corresponding changes in long-term mean winter temperatures (30-year means, 1973–2002) (c) are shown: 1 = North-Rhine Westphalia (DE), 2 = Bavaria (DE), 3 = Alsace (FR), 4 = Lower Austria (AT), 5 = Espace Mittelland (CH), 6 = Carinthia (AT).

In this study, we investigated the effect of seasonal mean temperatures and seasonal precipitation sums on the relative change in annual wild boar population size (i.e., rate of growth, λ) using long-term data on wild boar hunting bags from a major part of the wild boars’ European distribution range (Fig 1a; details in S1 Table). We further tested the effect of food availability on λ in a subsample of our data for which information on beech masting and areas under cultivation of corn and potatoes was available. We focussed on these fruits because they are known to play a major role in the food composition of wild boar [25].

We hypothesized that (1) major increases in the size of wild boar populations are linked to milder winter temperatures and changes in precipitation. If wild boar population dynamics are indeed affected by climate, particularly temperature, we envisioned two alternative scenarios: (i) There is a single critical species-specific winter temperature threshold for population growth for all European wild boar populations. (ii) Physiological trade-offs generate locally differing responses to ambient temperatures leading to population-specific winter temperature thresholds for population growth. (2) We further hypothesized that, in addition to direct climatic effects, increased abundance of food accelerates wild boar population growth by compensating the negative effects of severe winters.

## Materials and Methods

### Data collection

Long-term (15–150 years) annual wild boar hunting bag data were acquired for 69 regions from 12 European countries. The relative change in the hunting bag from one year to another (λ = bag_t_/bag_t-1_) was calculated after excluding hunting bags smaller than 0.01 animals shot/km² (799 data points), as such small values cause large uncertainty in estimating λ. This procedure yielded a data set in which most data points prior to 1950 were excluded, allowing us to focus mainly on the period showing exponential wild boar population growth (see Fig 1b for some examples). Further, 87 data points were excluded that could have been affected by the political situation at that time (World War 2, Balkan War, split of Czechoslovakia). The final data set included 64 regions out of 12 European countries (Fig 1a; details in S1 Table). In sum we analysed a total of 2075 annual hunting bags (mean: 32.5 years per region).

Monthly precipitation sums and mean temperatures were acquired for the respective regions and periods (S2 Table). Seasonal (3 months) precipitation sums and temperature means (weighted by the number of days per month) were calculated from the monthly data for spring (March–May), summer (June–August), autumn (September–November), and winter (December–February). Spatial autocorrelation analysis of the climatic data revealed that in none of the climatic variables the difference between climate stations increased below a distance of 300 to 400 km (S1 Fig). However, climate stations used were not separated by more than 268 km to the next climate station in the data set. Thus, climate data can be considered representative even for the largest regions in our data set.

Effects of food availability on population dynamics were investigated using the Austrian subsample (*n* = 135 years, 6 regions (S1 Table), mean: 22.5 years per region). Rank data on beech mast intensity (0 = < 30% pollination, mast failure; 1 = 30–50% pollination; 2 = 50–70% pollination; 3 = 70–85% pollination; 4 = 85–100% pollination, full mast) were obtained from the Austrian Federal Office and Research Centre for Forests (BFW, bfw.ac.at/rz/pollen.main) for 1976–2013. In years where multiple measurements were available per region the rank data were averaged in order to obtain a more accurate measurement for the whole region. Data on areas under cultivation of corn (*Zea mays* L.) and potatoes (*Solanum tuberosum* L.) were acquired from Statistics Austria (www.statistik.at) and were available from 1960 to 2011 and from 1966 to 2011, respectively.

Male and/or female adult (> 2 years) wild boar body mass data were obtained for one Italian region (Arezzo, Tuscany; M. Apollonio, pers. com.), three French regions [26, 27], three German regions [28], one Spanish region [29], one region from the Netherlands [30], three regions of the former Soviet Union, reviewed in [25], and one Austrian region (own data). It has been shown recently that in many species body size decreases as a response to climate change [31]. Consequently, in order to include only data for comparable periods, data published before 1970 were excluded from the analysis of body mass. If only dressed body mass was available, live body mass was estimated by multiplying dressed mass by 1.25 (for Central European data) and 1.15 (for Eastern European data), respectively, to account for different ways of dressing in these areas [25].

National data on the number of traffic accidents involving wild boar and the number of cars and trucks registered were acquired for Austria (1974–2011; Source: Statistics Austria, www.statistik.at), Germany (2007–2011; Sources: German Hunting Association, and German Federal Office of Statistics, www.destatis.de), and Switzerland (1992–2011; Source: Swiss Federal Office of Statistics, www.bfs.admin.ch). In these three countries every accident involving a game animal has to be reported to the police and is therefore well documented.

### Data analysis

To test the reliability of wild boar hunting bags as a proxy for wild boar population size, we computed Pearson’s correlation coefficients for the relationship between the number of traffic accidents involving wild boar (corrected for the number of cars and trucks registered) and the hunting bag (S2a–S2c Fig) as well as those between the relative year-to-year changes in the number of traffic accidents and λ (S2d–S2f Fig). This was done for Austria, Germany and Switzerland (i.e., countries where reliable traffic accident data were available), with the latter in parts employing a different hunting system (S2 Fig).

To test climatic effects on λ, temperature means and precipitation sums were considered during six seasons, spring (March-May), summer (June-August) and autumn (September-November) of the previous year, winter (December-February), and spring and summer of the census year. This large number of climatic variables and their interactions rendered statistical models computationally unfeasible. We therefore used the random forest method (a combination of binary decision trees created of bootstrapped samples of the data set and choosing randomly a subset of explanatory variables at each split [32, 33]) to pre-select important variables among all possible climatic variables. The number of variables randomly sampled as candidates at each split was set to 4 (number of predictor variables divided by 3) and the number of trees in the forest was set to 2000. The result of this analysis was very robust to changes of these parameters and showed that seasonal precipitation data contributed little to explaining the observed variance in λ (S3 Fig). Therefore, only seasonal temperature data were included in subsequent analysis. Additionally, regional long-term mean winter and summer temperatures as well as their interactions with current mean winter and summer temperatures were included in the model in order to test for the effect of long-term thermal conditions. We chose the 30-year period from 1973 to 2002 to calculate average long-term temperatures because data were available for all regions without missing values for that period. We further included the long-term average of summer and yearly precipitation sums in the model, which were calculated over the same period as the long-term temperatures. Further, to test whether population density affected λ, 5-year averages of hunting bags (animals shot per km^2^) preceding the census year were included in the model, as a proxy of density, along with its interaction with mean winter temperature. The use of hunting bag data as a proxy of density is justified by the strong correlation of hunting bag data and number of traffic accidents involving wild boar (see S2a–S2c Fig). The average of the previous 5-years was chosen instead of a single measure from the respective previous year to avoid “regression to the mean” effects [34], because the chance of a high λ occurring at random is much higher after a year with a low population density. To ease comparisons all variables were standardized to a mean of 0 and a SD of 0.5 [35]. Finally, country and region were included in all models as nested random effects in order to correct for potentially consistent regional influences on hunting bags (e.g. differences in overall hunting pressure). Inspection of the distribution of residuals of this full linear mixed effects (lme) model by means of histograms and quantile-quantile plots gave no evidence for serious deviations from normality. This also applies to all models described below. We used linear mixed effects models because the pre-analyses showed that those were superior in describing the data, according to the Akaike Information Criterium corrected for small sample size (AICc), compared to additive mixed effects models.

The full lme model was further analysed using a multi-model averaging approach [36]. Hereby, the multi-model average was calculated by including all possible nested models. However, the averaged estimates did not differ essentially when only certain subsets of models (i.e., with a delta AICc of below 4 and 10, respectively) were used for calculating the model-averaged parameter estimates (data not shown). Model-averaged parameter estimates were calculated with the natural average method, in order to avoid shrinkage towards zero, especially of weak effects [35, 37]. As we further aimed to determine which variable had the strongest impact on λ we also calculated model averaged estimates using the zero-method [35]. However, the order of effect sizes of significant variables was identical for both model-averaging methods (compare Table 1 vs. S3 Table). To obtain correct parameter estimates for main effects of variables involved in interactions, we selected models used for averaging as follows: If an interaction was significant in the model-average we selected only models where this interaction also was significant to calculate the weighted averages of the parameter estimates (i.e., annual and long-term winter temperature; Table 1a). In contrast, if an interaction was not significant in the model-average, we selected only models not containing this interaction, and models in which this interaction was not significant, to calculate weighted averages (i.e., annual and long-term summer temperature, and density; Table 1). This procedure of averaging also was applied in all further multi-model averages described below. Model-averaged residuals were subsequently checked for spatio-temporal autocorrelation by means of semivariograms which showed that residuals were neither temporally nor spatially autocorrelated (Figures A and B in S1 File).

**Table 1:** Impact of climate and food availability on wild boar population growth rate (λ)

(a) European data set (*n* = 2075)					(b) Austrian data set (*n* = 135)				
term	estimate	SE	*P*	RVI	term	estimate	SE	*P*	RVI
**winter temperature**	**0.180**	**0.025**	**< 0.001**	**1.00**	**winter temperature**	**0.237**	**0.064**	**< 0.001**	**1.00**
**long-term winter temperature**	**-0.252**	**0.033**	**< 0.001**	**1.00**	**mast**	**0.284**	**0.112**	**0.012**	**1.00**
**prior autumn temperature**	**0.179**	**0.027**	**< 0.001**	**1.00**	**mast: winter temperature**	**-0.926**	**0.247**	**< 0.001**	**0.98**
**population density**	**-0.065**	**0.016**	**< 0.001**	**1.00**	**prior autumn temperature**	**0.173**	**0.062**	**0.005**	**0.94**
**summer temperature**	**-0.050**	**0.023**	**0.031**	**0.82**	**long-term winter temperature**	**-0.172**	**0.084**	**0.044**	**0.82**
**winter temperature: long-term winter temperature**	**-0.059**	**0.027**	**0.030**	**0.80**	population density	-0.133	0.113	0.24	0.50
					area under cultivation of potatoes	-0.096	0.071	0.18	0.45
prior spring temperature	0.039	0.023	0.09	0.62	area under cultivation of corn	-0.092	0.088	0.30	0.38
prior summer temperature	-0.027	0.026	0.29	0.41	summer temperature	-0.063	0.061	0.31	0.34
long-term summer temperature	-0.012	0.029	0.67	0.39	prior spring temperature	-0.022	0.055	0.70	0.25
long-term summer precipitation	0.021	0.024	0.40	0.37	winter temperature: long-term winter temperature	-0.045	0.104	0.67	0.21
winter temperature: population density	0.029	0.033	0.37	0.37					
long-term yearly precipitation	0.012	0.025	0.65	0.34	mast: long-term winter temperature	-0.098	0.664	0.88	0.19
spring temperature	-0.011	0.025	0.64	0.30	mast: population density	-0.097	0.291	0.74	0.13
summer temperature: long-term summer temperature	-0.022	0.029	0.44	0.10					

Parameters estimate, standard error (*SE*), p-value (*P*), and relative variable importance (*RVI*) for each term included in the multi-model averages of the European data set (a) and the Austrian subset (b). Interaction terms are indicated by colons, significant predictor variables are highlighted bold. See methods for the definition of seasons.

Due to smaller sample size only those variables were included in the full lme model of the Austrian multi-model average that were found to be important in the European wide analysis (Table 1a). To assess effects of food availability, beech mast of the previous year (binary coded; 1: moderate or full beech mast (categories 3 or 4; pollination ≥ 70%), 0: less than moderate beech mast (categories 0–2; pollination < 70%), as well as areas under cultivation (% of total area of a region) of corn and potatoes during the previous year were included. Furthermore, the interaction between beech mast and winter temperature was included, because the vast amount of energy provided by tree seeds might have compensated for unfavourable climatic conditions. In addition to region, beech mast in the respective census year was added as a random factor to the Austrian subset models, to account for possible effects of mast intensity on hunting success.

Further, we tested whether the frequencies of mast failure (mast category ≤ 1), moderate (1 < mast category ≤ 3) and full beech mast (mast category > 3; each coded 1/0) have changed in Austria since 1976 by computing three binomial linear mixed effects models, respectively, each with year as fixed and region as random effect (*n* for all three models = 165 years, 6 regions, mean: 27.5 years per region).

In order to test for an association of body mass with regional climate we computed the multi-model average of a linear model with adult (> 2 years) wild boar body mass as a function of the long-term mean winter-temperature (see below), sex and their interaction (*n* = 22 European regions).

All statistical analyses were performed in R.3.0.2 [38]. Linear mixed-effects models were computed using the package lme4 [39], for multi-model averaging we used the package MuMIn [40] and own functions. The random forest analysis was performed using the package randomForest (V 4.6–10) [41]. Spatio-temporal autocorrelation of the residuals was checked using the package gstat [42].

## Results

### Validity of hunting bag data

We observed strong correlations between hunting bags and the number of traffic accidents involving wild boar (corrected for the number of cars registered) in Austria, Germany, and Switzerland (Austria (AT): *r* = 0.96, *P* < 0.001; Germany (DE): *r* = 0.99, *P* < 0.001; Switzerland (CH): *r* = 0.84, *P* < 0.001; S2a–S2c Fig). Further, we found similarly strong correlations between the λ calculated from hunting bags and year-to-year change in car accidents involving wild boars in Austria, Germany, and Switzerland (AT: *r* = 0.73, *P* < 0.001; DE: *r* = 0.99, *P* < 0.001; CH: *r* = 0.90, *P* < 0.001; S2d–S2f Fig). These strong correlations indicate that hunting bags are a reliable proxy for changes in population size, as car accidents represent a random sample [43].

### Climatic effects

As determined by our initial analysis using the random-forest approach (see Materials and Methods), seasonal precipitation data had no significant effect on wild boar population growth (S3 Fig). Long-term summer precipitation and long-term yearly precipitation also had no effect on λ (Table 1a).

**Fig 2:**
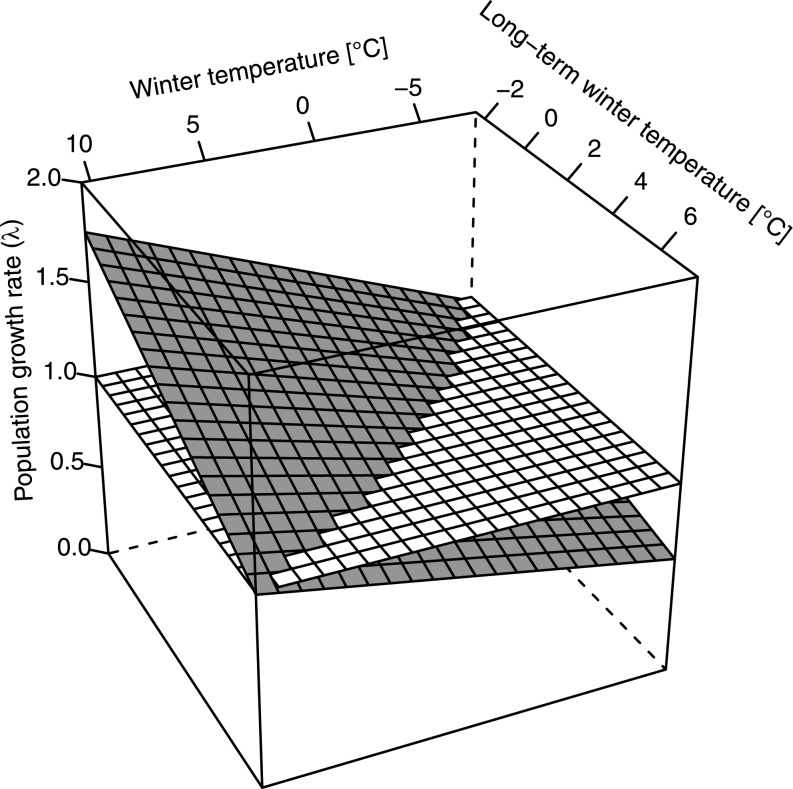
European-wide effect of winter temperature on subsequent wild boar population growth (λ). Long-term mean winter temperature refers to the 30-year average of winter temperature (December to February) from 1973 to 2002 and indicates how cold or warm a specific region in general is. The grey plane shows model predictions for λ, the white plane represents λ = 1, i.e., no change in population size.

**Fig 3:**
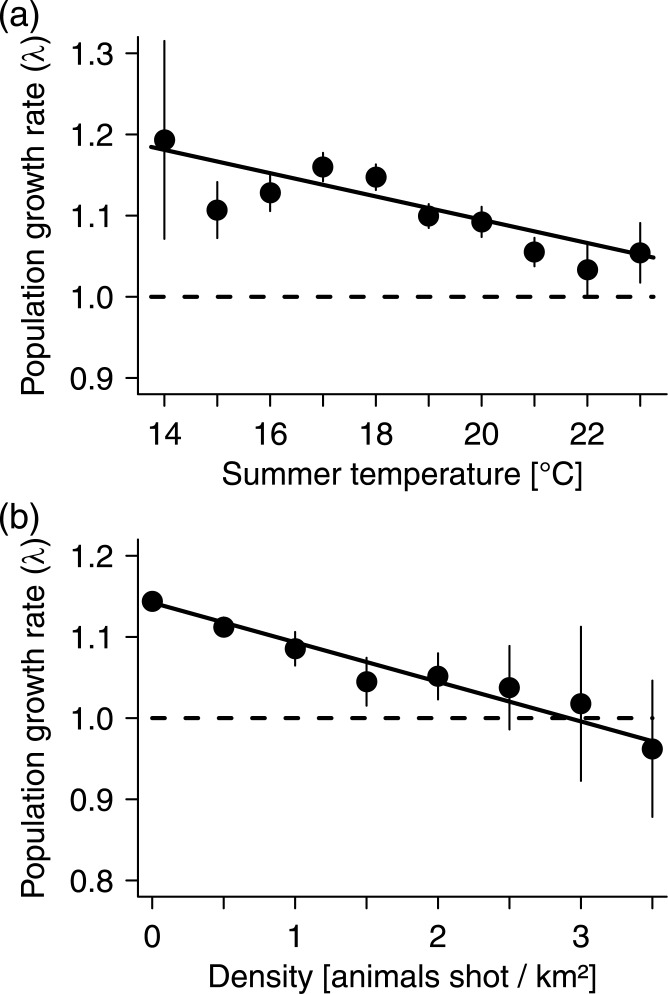
Effect of (a) summer temperature and (b) previous population density on wild boar population growth (λ). Means and standard errors of the means for bins of summer temperature (ranges of 1 degree) and density (ranges of 0.5 animals shot per km²) are plotted with the model prediction of the respective variable.

Population growth in European wild boars was positively affected by annual and negatively by long-term winter temperature (Table 1a, Fig 2). Importantly, these two variables showed a significant interaction (Table 1a, Fig 2), indicating that wild boar populations in cooler regions were more strongly affected by an increase in annual winter temperature than populations in warmer regions (Fig 2). Additionally, this interaction reflected that populations began to grow (λ > 1) already at much lower winter temperatures in cooler regions compared to warmer areas (Fig 2). Further, autumn temperature of the previous year had a strong positive effect on population growth (Table 1a), in contrast to summer temperature in the census year, which had a much weaker negative effect (Table 1a, Fig 3a). We found no significant effect of long-term summer temperature on λ (Table 1a).

Apart from climatic variables, population density, as indicated by the mean hunting bag during the 5 years prior to the census, had also a slightly negative effect on λ (Table 1a, Fig 3b). However, there was no significant interaction between density and winter temperature (Table 1a), indicating that density effects did not differ between warmer and colder regions.

### Food availability and mast frequency

**Fig 4:**
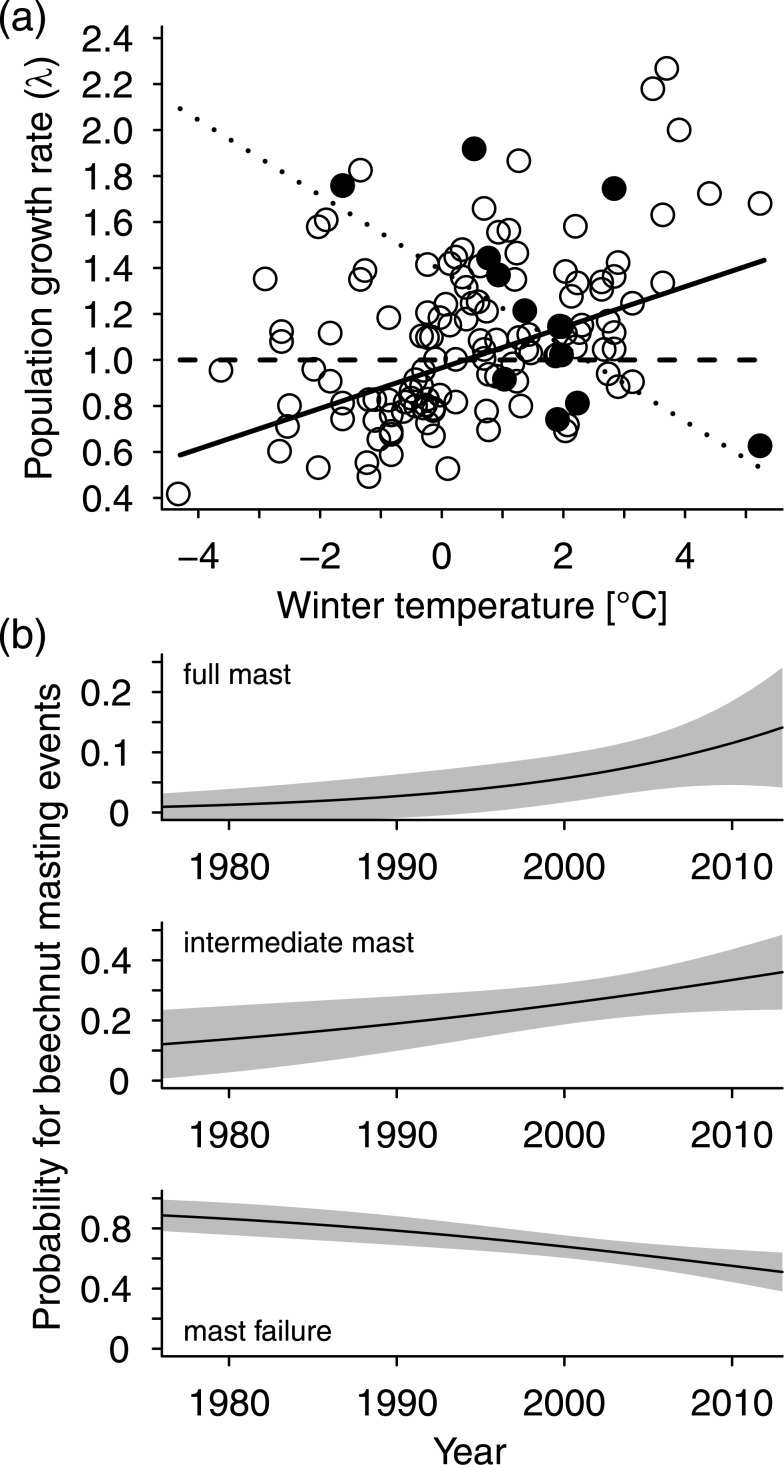
Wild boar population growth and beech masting. (a) The effect of winter temperature on population growth rate (λ) in years after full or moderate mast (•, ···), and in years after mast failure (◦, ―). The horizontal dashed line indicates no change, i.e. λ = 1. (b) Probability of beech mast events in Austria from 1976 to 2013. Lines represent predicted values from binomial models (see Methods for details), shaded areas 95% confidence limits.

The effect of beech masting events and other food sources was explored in the Austrian subset of our data. We found that moderate or full mast in the previous autumn, i.e., high food availability during winter, completely outweighed the negative effects of cold winter conditions on population growth (Table 1b, Fig 4a). There was no significant interaction between beech mast and population density or beech mast and long-term winter temperature (Table 1b). Further, we did not find any effects of areas under cultivation of corn and potatoes on population growth (Table 1b).

The frequency of mast failure years in Austria decreased significantly since 1976 (slope = -0.054, se = 0.019, *P* = 0.004, Fig 4b). Accordingly, both intermediate masting events (slope = 0.038, se = 0.020, *P* = 0.052, Fig 4b) and full masting events (slope = 0.077, se = 0.040, *P* = 0.053, Fig 4b) showed strong trends of increase.

### Body mass

**Fig 5:**
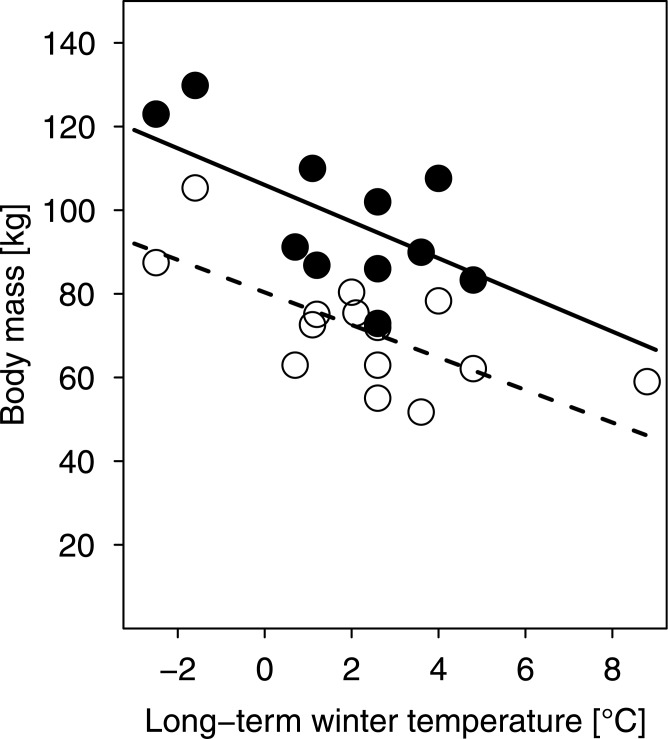
Body mass of adult wild boar in relation to local long-term winter temperatures. Males (•, ―), females (◦, - -), long-term mean winter temperature is for each region the 30-year average of December to February from 1973 to 2002.

Adult wild boar body mass correlated negatively with long-term (30-year) regional mean winter temperature (slope = -3.9, se = 1.1, *P* < 0.001; Fig 5). Males and females showed different intercepts (estimate = 25.7, se = 5.6, *P* < 0.001; Fig 5) but no difference concerning the slope of the relationship (*P* = 0.38; Fig 5). Wild boars in the coldest region were about 30–40 kg (~ 30%) heavier than in the warmest region investigated.

## Discussion

It has been shown in previous studies that ungulate traffic collisions in Europe accurately reflect the population size and therefore can be regarded as random sampling [43, 44]. The strong correlations between the relative year-to-year changes in the number of traffic accidents and the λ calculated from hunting bags in all regions investigated (S2d–S2f Fig) show that fluctuations in hunting bags largely reflect those in population size and, consequently, are a good proxy for relative changes in wild boar population size, at least at the coarse scales considered here.

Our analysis shows that wild boars are apparently highly susceptible to cold winter conditions (Fig 2), as those were consistently followed by population declines. Cool autumns also had a negative impact on population growth, which may simply reflect an early onset of winter. It is known that cold winters lead to increased juvenile mortality [43], which is a major driver of wild boar population dynamics [18]. Susceptibility to low winter temperatures can also explain why wild boars occur at higher densities in warmer regions [45]. Of course, we cannot exclude that other factors such as changes in agricultural practices or changes in hunting pressure [21, 46, 47] also influence growth of wild boar populations. However, although it was reported that hunting pressure affects life-history traits and demography in the wild boar [21, 46, 47], no study so far has actually shown that population growth rate in wild boar is significantly affected by hunting pressure. Moreover, a recent study underlines that recreational hunting has no influence on wild boar population growth [15]. Wild boar populations across Europe have been growing irrespective of whether the number of hunters have increased, decreased or remained stable [15]. Furthermore, even though such factors may well have contributed to the considerable noise in λ, they were not strong enough to mask the dominant effect of winter temperature. Increasingly milder winters as a result of climate change (Fig 1c; see also [48]) must therefore be considered as a major reason for the European-wide massive increase of wild boar during the last decades (Fig 1b).

Flexible and generalist species like the wild boar are generally more likely to cope with or even benefit from climate change compared to specialist and less flexible species [2]. The positive effect of warming climate on wild boar population growth identified here, together with results from previous studies on other ungulates [10, 49, 50], is in line with this. Population size is positively associated with milder winters and an early onset of spring in red deer (*Cervus elaphus* L.) [49, 50], an ungulate species with flexible birth dates [51]. In contrast, in roe deer (*Capreolus capreolus* L.), where females have inflexible birth dates, an earlier onset of spring has a negative impact on population growth [10]. The latter is apparently caused by an increasing phenological mismatch between onset of vegetation growth and birth [10]. Furthermore, being an income breeder [52], the roe deer can be expected to be more severely affected by such a phenological mismatch than wild boar or red deer.

Surprisingly, climate change associated wild boar population increases were not restricted to the coldest regions. In fact, we show for the first time that a positive relation between winter temperature and wild boar population growth exists over a large climatic range, but the effect is weaker in warmer regions. Further, in warmer areas there was a higher threshold-temperature for the onset of population growth (Fig 2). Therefore, population-specific temperature thresholds can explain why changes in average winter temperature have led to almost concurrent increases in wild boar population sizes all over Europe (Fig 1a and 1b; [14]), despite differences in local climates.

The lack of a common threshold winter temperature initiating population growth could be either due to local adaptation, phenotypic plasticity, or to a density-climate interaction, as population densities in cooler regions are generally lower [45]. Density-dependent mechanisms have been shown to affect life-history traits and consequently population growth in several other ungulate species [50, 53–57]. However, the effect of density in the present analysis was relatively small compared with the impact of long-term winter temperature (Table 1a). Additionally, our results gave no evidence for a significant interaction of density with annual winter temperature (Table 1a), which might have been expected because environmental fluctuations are likely to affect animals more strongly at higher population densities [55, 57]. Further, as yet there is no flattening of the exponential population growth curves (see Fig 1b for some examples), and a previous study indicates that density dependence in general plays a minor role in wild boar population dynamics [58]. Overall, this suggests that the locally differing temperature-thresholds for the onset of population growth identified here are probably not caused by a density-climate interaction but are rather due to phenotypic plasticity or local adaptation. With our data, we cannot differentiate between these two mutually not exclusive alternatives. However, wild boar populations in Europe have been shown to be genetically distinct [17], rendering at least some degree of genetic adaptation likely. Such genetic differentiation of populations and adaptation to local climatic conditions has been shown in several fish species, e.g. [5, 59], whereas changes in body size of red-billed gulls (*Chroicocephalus scopulinus* F.) and great tits (*Parus major* L.) in response to climate change seemed to be due to phenotypic plasticity [8, 9].

If differences in the local responses to climate in wild boar are due to local adaptation or phenotypic plasticity, one would expect regional clines in morphological or physiological characteristics. One important trait to consider in this context is body size. Body size is known to be a major factor affecting thermoregulation, energy requirements, and ultimately survival in different climates by changing the surface to volume ratio and thus relative heat loss (Bergmann’s rule [7]). Our results confirm previous indications for the applicability of Bergmann’s rule to the wild boar, e.g. [60], as animals of this species in colder regions are indeed heavier (Fig 5). While larger body size in cool climates reduces the relative heat loss, small body size in warmer regions, on the other hand, leads to lower total metabolic rates and higher rates of heat loss. Hence, smaller body size may help wild boar to withstand high summer temperatures despite its limited ability to use evaporative cooling, and potentially serve to reduce reproductive constraints due to the limits of heat dissipation during lactation [61]. However, smaller body size does not seem to entirely offset adverse effects of heat, as underlined by our finding that wild boar population growth generally decreased, though only slightly, with higher summer temperatures. Consequently, it seems plausible that regionally differing body mass of wild boars, no matter whether it is due to local adaptation or a common reaction norm, evolved because of trade-offs between the benefits of large size in winter and of small size in summer. These locally different phenotypes in turn could have caused the region-specific temperature thresholds observed here.

Several studies on birds, e.g. [8], and mammals, e.g. [62, 63], showed that the average body size in populations often changes in response to climate change. So far it is not known whether this is also the case in the wild boar and how this might affect the impact of rising temperatures on wild boar populations. This question needs to be targeted in further studies.

The negative effect of summer temperature on wild boar population growth, however, was very small in the European-wide data set (Table 1a) and even absent in the Austrian subset (Table 1b). Therefore, higher than average summer temperatures probably play an important role only in specific habitats, whereas warm and rainy summers may even have a positive effect on reproduction in certain populations [21]. It remains unclear however, which habitats that might be as the effect of summer temperature, in contrast to winter temperature, was apparently independent of the local long-term climate (Table 1a).

In addition to these direct abiotic effects of climate change on wild boar populations we also identified indirect effects via food availability. Beech nuts and acorns are the most important food resource for wild boar [25, 64] but seed production in these species is highly variable and can cease entirely over large areas. This mechanism is commonly viewed as a strategy of trees to swamp seed-predators at unpredictable intervals [65]. Our analysis showed that the frequency of beech masting years has increased over the last decades (Fig 4b), presumably due to climate change [24]. This finding, together with the fact that cold winters had no negative effect on population growth when food resources were abundant, shows that the effect of climate change on population growth of wild boar is two-fold: Cold winters have become rarer and, on top of this, the remaining severe winters became increasingly ineffective in diminishing wild boar populations because of the increasing frequency of masting years. In such years, beech or oak trees produce vast amounts of energy rich seeds that are available from autumn until spring in the following year [18, 23]. If abundant, this food source likely enables juveniles to cope even with high thermoregulatory costs in a severe winter, and adults to accumulate high amounts of body energy reserves for reproduction in the following year. This result also indicates that low survival in cold winters is apparently not caused by a limited thermogenic capacity. Instead, increased winter mortality seems to be caused by a negative energy balance, i.e., when high thermoregulatory costs, due to severe cold, cannot be matched by the available food, especially when high caloric seeds are absent.

In addition to tree seeds, increased crop availability in a human-shaped landscape has previously been assumed to provide an important source of energy for wild boars [23]. However, the lack of significant effects of areas under cultivation of corn and potatoes on wild boar population dynamics indicates that this is apparently not the case, presumably because significant quantities of these crops are available only during a short period prior to harvesting.

## Conclusions

It has been shown recently that, even for closely related species with similar ecological niches, responses to climate change cannot be extrapolated easily from one species to another [66]. Our results show for the first time that this holds even for separate populations within a species and therefore questions any predictions of the consequences of climate change for population dynamics if they are based on responses to environmental factors, such as ambient temperature, in a single or just a few local populations. Our results further underline the need to take indirect ecological effects into account, such as increasing food availability (e.g. accelerated tree-masting frequency), when studying the impact of climate change on species or ecosystems.

## Supporting Information

S1 Fig Semivariogram showing the spatial autocorrelation structure of the climatic variables. (PDF)

S2 Fig Wild boar hunting bags and traffic accidents involving wild boar. Comparison between annual wild boar hunting bags and the number of traffic accidents involving wild boar as well as between the relative change in the hunting bag and the relative change in traffic accidents. The illustrated findings justify the use of wild boar hunting bags as a proxy for population size, since traffic accidents can be regarded as a random sampling of population size.
(PDF)

S3 Fig Importance of climatic variables according to a ‘random forest’ analysis. (PDF)

S1 Table Overview over the wild boar hunting bag data from all regions included in the analysis. For each region the area [km²], the earliest and the latest hunting bag data point, the minimum and the maximum hunting bag, the long-term (1973–2002) winter mean temperature is shown, as well as sources of the hunting bag data.
(PDF)

S2 Table Overview over the climatic data from all regions included in the analysis. For each region the number and the name of the climate station used are given as well as the latitude, longitude, and altitude of the respective station. Further, the year of the oldest data point for monthly mean temperature and monthly precipitation sum is given as well as the source of the data.
(PDF)

S3 Table Model-averaged parameters estimates and standard errors for the European and the Austrian multi-model average are given. These results have been obtained using the zero method and are given in comparison to Table 1 showing the results obtained with the natural-average method.
(PDF)

S1 File Spatial and temporal autocorrelation analysis of the residuals of the European multi-model average, including the spatio-temporal semivariogram of the residuals (Figure A) and the bootstrapped spatial autocorrelation structure of the residuals (Figure B). (PDF)
