# Supplementary material for: What Is a Mild Winter? Regional Differences in Within-Species Responses to Climate Change
Source: PLoS One. 2015 Jul 9;10(7):e0132178. doi: 10.1371/journal.pone.0132178 (PMC4497731; doi:10.1371/journal.pone.0132178)
Supplement: S1 Fig — (PDF) [file pone.0132178.s001.pdf]

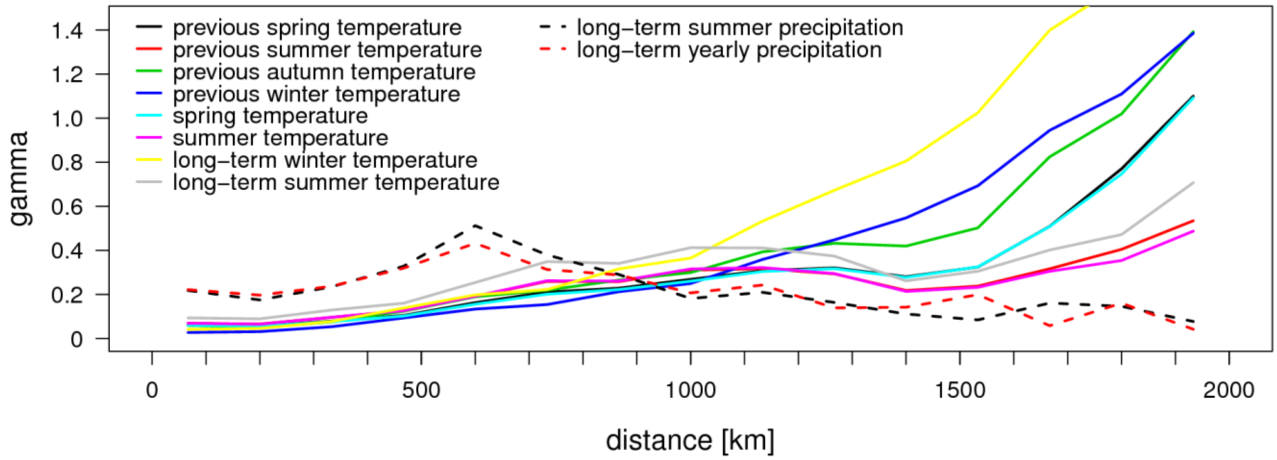

S1 Fig. Semivariogram showing the spatial autocorrelation structure of the climatic variables.
