# Supplementary material for: What Is a Mild Winter? Regional Differences in Within-Species Responses to Climate Change
Source: PLoS One. 2015 Jul 9;10(7):e0132178. doi: 10.1371/journal.pone.0132178 (PMC4497731; doi:10.1371/journal.pone.0132178)
Supplement: S2 Fig — Comparison between annual wild boar hunting bags and the number of traffic accidents involving wild boar as well as between the relative change in the hunting bag and the relative change in traffic accidents. The illustrated findings justify the use of wild boar hunting bags as a proxy for population size, since traffic accidents can be regarded as a random sampling of population size. (PDF) [file pone.0132178.s002.pdf]

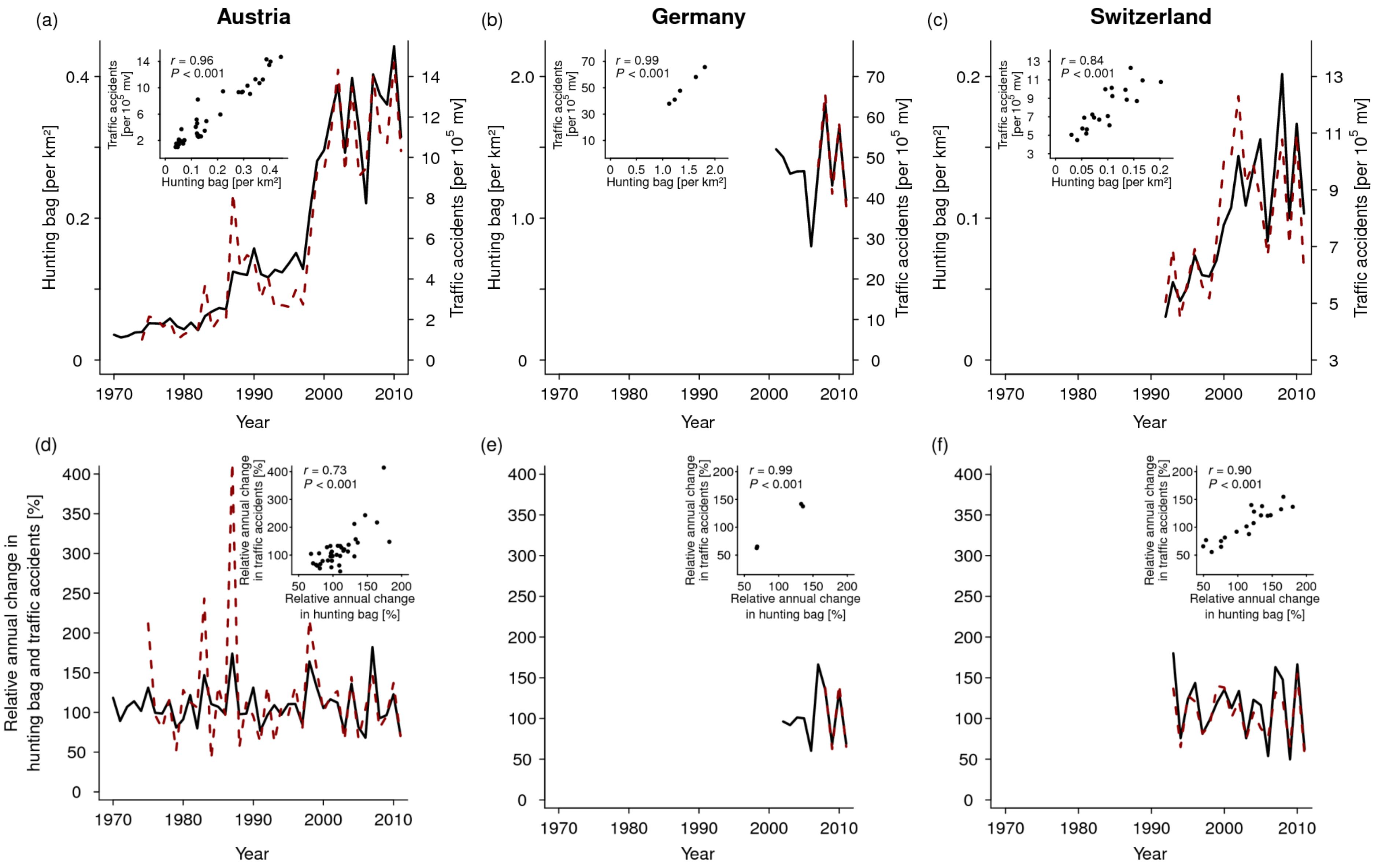


**S2 Fig. Wild boar hunting bags and traffic accidents involving wild boar**. Data are shown for (a, d) Austria (b, e) Germany, and (c, f) Switzerland. In a, b, c — shows the hunting bag (left axis) and - - shows the traffic accidents corrected for the number of motor-vehicles (mv = cars and trucks) registered (right axis). The insert plots show the correlations between these two variables. In d, e, f — shows the relative change in the hunting bag and - - shows the relative change in traffic accidents (also corrected for the number of motor-vehicles registered). In both analyses (a-c vs. d-f) very high correlation coefficients were obtained for all three countries, despite the fact that there is a different hunting system in some parts of Switzerland. These findings justify the use of wild boar hunting bags as a proxy for population size, since traffic accidents can be regarded as a random sampling of population size.
