# Supplementary material for: What Is a Mild Winter? Regional Differences in Within-Species Responses to Climate Change
Source: PLoS One. 2015 Jul 9;10(7):e0132178. doi: 10.1371/journal.pone.0132178 (PMC4497731; doi:10.1371/journal.pone.0132178)
Supplement: S3 Fig — (PDF) [file pone.0132178.s003.pdf]

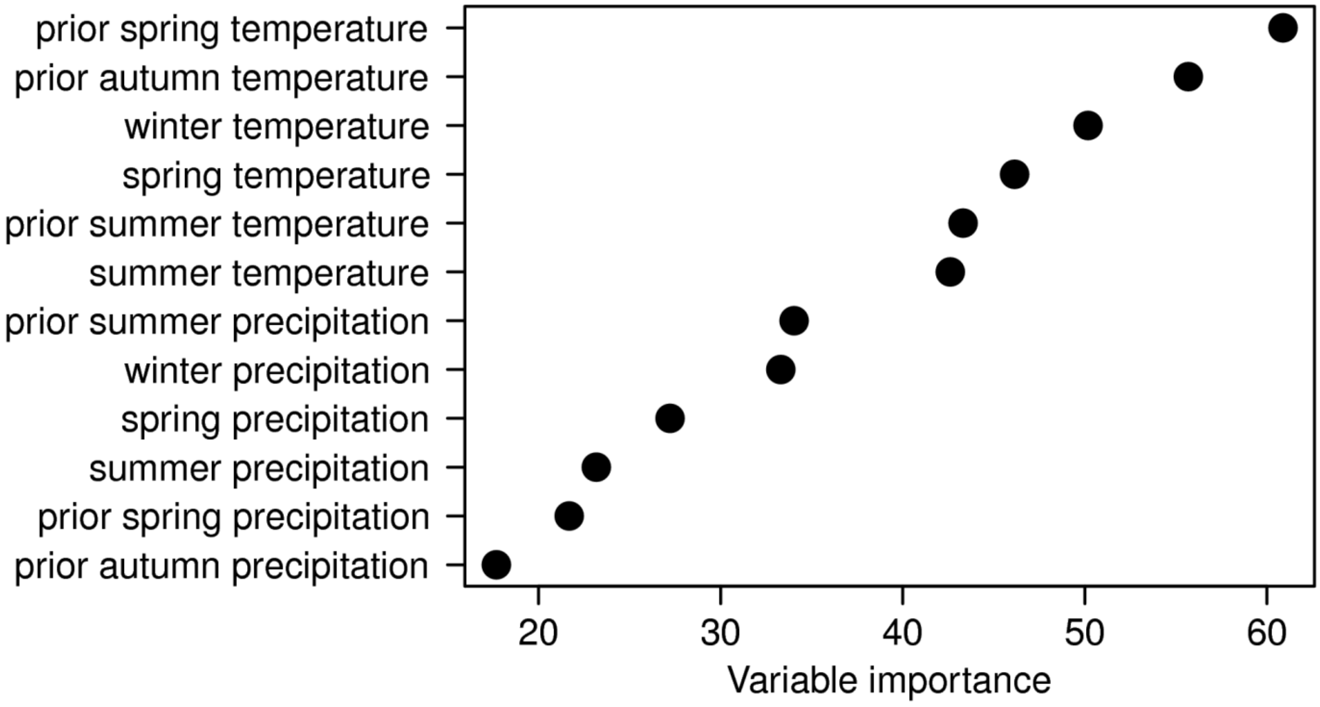

S3 Fig. Importance of climatic variables according to a 'random forest' analysis. Variable importance is given as the percental increase of the mean standard error under random permutation of the respective variables. See methods for the definition of seasons.
