# Supplementary material for: What Is a Mild Winter? Regional Differences in Within-Species Responses to Climate Change
Source: PLoS One. 2015 Jul 9;10(7):e0132178. doi: 10.1371/journal.pone.0132178 (PMC4497731; doi:10.1371/journal.pone.0132178)
Supplement: S1 Table — For each region the area [km²], the earliest and the latest hunting bag data point, the minimum and the maximum hunting bag, the long-term (1973–2002) winter mean temperature is shown, as well as sources of the hunting bag data. (PDF) [file pone.0132178.s004.pdf]

S1 Table. Overview over the wild boar hunting bag data.

Country	Region	area	earliest	latest	min	max	winter	Source
Austria	Burgenland	3,966	1931	2011	0.012	2.375	0.7	Stat. Austria
Austria	Carinthia	9,536	1874	2011	0.016	0.039	-2.3	Schwenk, S. (1985); Stat. Austria
Austria	Lower Austria	19,178	1868	2011	0.012	1.207	1.0	Schwenk, S. (1985); Stat. Austria
Austria	Styria	16,392	1874	2011	0.013	0.127	0.0	Schwenk, S. (1985); Stat. Austria
Austria	Upper Austria	11,982	1874	2011	0.011	0.075	0.4	Schwenk, S. (1985); Stat. Austria
Austria	Vienna	415	1935	2011	0.080	5.465	1.0	Stat. Austria
Belgium	Wallonia	16,844	1974	2011	0.293	1.442	2.4	FAB
Croatia	-	56,538	1972	1990	0.119	0.803	2.4	Schwenk, S. (1984)
Czech Republic	-	7,969	1902	1977	0.010	0.796	-0.6	Schwenk, S. (1985); Bartos (pers. com.)
France	Alsace	14,522	1884	2011	0.029	4.702	2.3	Schwenk, S. (1982); ONCFS
France	Aquitaine	41,309	1975	2011	0.017	0.679	5.9	ONCFS
France	Auvergne	26,013	1975	2011	0.052	0.514	4.4	ONCFS
France	Brittany	27,209	1976	2011	0.010	0.150	5.9	ONCFS
France	Burgundy	31,582	1975	2011	0.049	1.059	2.8	ONCFS
France	Centre	39,151	1975	2011	0.065	1.348	4.1	ONCFS
France	Champagne-Ardenne	25,606	1975	2011	0.111	1.671	3.5	ONCFS
France	Corsica	8,680	1980	2011	0.945	3.165	8.9	ONCFS
France	Franche-Comté	16,202	1977	2011	0.070	0.985	2.6	ONCFS
France	Île-de-France	11,494	1977	2011	0.074	1.570	4.4	ONCFS
France	Languedoc-Roussillon	27,376	1977	2011	0.348	2.505	6.7	ONCFS
France	Limousin	16,942	1975	2011	0.016	0.743	4.6	ONCFS
France	Lorraine	23,547	1975	2011	0.126	2.636	2.6	ONCFS
France	Lower Normandy	17,589	1975	2011	0.070	0.976	5.6	ONCFS
France	Midi-Pyrénées	45,348	1975	2011	0.036	0.824	6.3	ONCFS
France	Nord-Pas-de-Calais	12,414	1975	2011	0.011	0.385	3.8	ONCFS
France	Pays de la Loire	32,082	1977	2010	0.014	0.385	5.6	ONCFS
France	Picardy	19,399	1977	2011	0.040	0.935	3.9	ONCFS
France	Poitou-Charentes	25,809	1975	2011	0.015	0.427	6.3	ONCFS
France	Provence-Alpes-Côte d'Azur	31,400	1977	2011	0.229	1.532	4.3	ONCFS
France	Rhône-Alpes	43,698	1975	2011	0.092	1.215	3.2	ONCFS
France	Upper Normandy	12,318	1975	2011	0.026	0.406	4.0	ONCFS

S1 Table (continued). Overview over the wild boar hunting bag data.

Country	Region	Area	earliest	latest	min	max	winter	Source
Germany	Baden-Württemberg	35,751	1956	2010	0.038	1.453	2.1	DJV
Germany	Bavaria	70,553	1909	2010	0.010	0.882	-0.3	Schwenk, S. (1983); DJV
Germany	Brandenburg & Berlin	30,370	1984	2010	1.273	2.752	1.2	DJV
Germany	Hesse	21,114	1953	2010	0.085	3.691	2.2	DJV
Germany	Lower Saxony & Bremen	48,004	1958	2010	0.066	1.200	2.0	DJV
Germany	Mecklenburg-Vorpommern	23,167	1984	2010	1.121	3.275	1.7	DJV
Germany	North Rhine-Westphalia	34,070	1953	2010	0.056	1.258	3.4	DJV
Germany	Rhineland-Palatinate & Saarland	22,416	1955	2010	0.120	3.866	2.1	DJV
Germany	Saxony	18,338	1984	2010	0.677	1.850	1.0	DJV
Germany	Saxony-Anhalt	20,443	1984	2010	0.810	1.767	1.1	DJV
Germany	Schleswig-Holstein & Hamburg	16,486	1956	2010	0.039	0.987	1.5	DJV
Germany	Thuringia	16,175	1984	2010	0.656	1.981	0.3	DJV
Hungary	Central Hungary	14,839	1962	2002	0.018	0.684	0.6	OVA
Hungary	historical Hungary	93,036	1896	1909	0.032	0.064	0.6	Schwenk, S. (1985)
Hungary	Northern Hungary	13,428	1974	1992	0.102	0.635	-1.1	OVA
Hungary	Northern Transdanubia	18,662	1962	2002	0.047	1.535	0.2	OVA
Hungary	Southern Transdanubia	17,953	1962	2002	0.032	1.719	0.9	OVA
Hungary	Trans-Tiszanian Region	27,623	1981	2002	0.012	0.116	-0.4	OVA
Luxemburg	-	2,586	1950	2003	0.085	1.842	1.3	ANF
Netherlands	Veluwe	621	1978	2011	0.628	8.744	3.6	Spek (pers. com.)
Poland	Central Poland	53,777	1977	2011	0.068	0.306	-1.2	GUS; Fruziński, B. (1992)
Poland	Eastern Poland	74,866	2001	2011	0.067	0.280	-0.9	GUS; Fruziński, B. (1992)
Poland	Northern Poland	60,455	2001	2011	0.410	0.852	-1.1	GUS; Fruziński, B. (1992)
Poland	Northwestern Poland	66,707	1977	2011	0.334	1.147	-0.2	GUS; Fruziński, B. (1992)
Poland	Podlaskie	20,187	1977	2011	0.069	0.309	-2.5	GUS; Fruziński, B. (1992)
Poland	Southern Poland	27,516	1977	2011	0.076	0.389	-1.3	GUS; Fruziński, B. (1992)
Poland	Southwestern Poland	29,359	1977	2011	0.285	1.132	0.2	GUS; Fruziński, B. (1992)
Slovakia	-	49,034	1894	1971	0.017	0.076	0.4	Findo (pers. com.)

S1 Table (continued). Overview over the wild boar hunting bag data.

Country	Region	Area	earliest	latest	min	max	winter	Source
Switzerland	Eastern Switzerland	12,225	1952	2011	0.016	0.086	0.2	FOEN
Switzerland	Espace Mittelland	4,621	1979	2011	0.027	0.316	0.8	FOEN
Switzerland	Lake Geneva region	8,719	1977	2011	0.013	0.089	0.7	FOEN
Switzerland	Northwestern Switzerland & Zurich	9,129	1985	2011	0.014	0.363	1.1	FOEN
Switzerland	Ticino	991	1990	2011	0.146	1.738	4.0	FOEN

For each region the area (*Area*) [km²], the earliest (*earliest*) and the latest (*latest*) hunting bag data point, the minimum (*min*) and the maximum (*max*) hunting bag [animals shot/km²] as well as the 30-year (1973-2002) winter mean temperature [°C] (*winter*) is shown. The last column specifies the sources of the hunting bag data. The data were acquired from the Federal Office for the Environment, Switzerland (FOEN, www.wild.uzh.ch/jagdst/), the Research Institute for Nature and Forest, Flanders, Belgium (INBO, through T. Scheppers), the Forestry Administration, Belgium (FAB, through A. Licoppe), the Nature and Forestry Agency, Luxemburg (ANF, through S. Cellina), the National Office for Hunting and Wildlife, France (ONCFS), the Annual Handbook of the German Hunting Association (DJV), Statistics Austria (Stat. Austria, www.statistik.at), and the Central Statistical Office, Poland (GUS, www.stat.gov.pl), and the National Game Management Database, Hungary (OVA, www.vvt.gau.hu). Further data for the Czech-Republic, the Netherlands, and Slovakia were kindly provided to us by Bartos, Spek, and Findo, respectively (all pers. com.). Historical data were taken from Schwenk [1-5] and Fruziński [6].

References

1. Schwenk S. Bayerische Jagdstatistiken von 1827 bis 1936. Lindner K, Schwenk S, editors. Bonn: Rudolf Habelt; 1983.

2. Schwenk S. Österreichische Jagdstatistiken von 1850 bis 1936. Bonn: Rudolf Habelt; 1985. 203 p.

3. Schwenk S. Jagdstatistiken Elsaß-Lothringen von 1882 bis 1920. Bonn: Rudolf Habelt; 1982.

4. Schwenk S, Ratzeburg JTC. Jagdstatistik des zollvereinten und nördlichen Deutschlands 1858/1868. Bonn: Rudolf Habelt; 1984.

5. Schwenk S. Jagdstatistiken der kleineren deutschen Staaten von der Mitte des 18. bis zum Anfang des 20. Jahrhunderts. Bonn: Rudolf Habelt; 1984.

6. Fruziński B. Dzik [Wild boar]. Warszawa: Wydawnictwo Cedrus; 1992.
