# Supplementary material for: What Is a Mild Winter? Regional Differences in Within-Species Responses to Climate Change
Source: PLoS One. 2015 Jul 9;10(7):e0132178. doi: 10.1371/journal.pone.0132178 (PMC4497731; doi:10.1371/journal.pone.0132178)
Supplement: S2 Table — For each region the number and the name of the climate station used are given as well as the latitude, longitude, and altitude of the respective station. Further, the year of the oldest data point for monthly mean temperature and monthly precipitation sum is given as well as the source of the data. (PDF) [file pone.0132178.s005.pdf]

S2 Table. Overview over the climatic data.

Country	Region	No.	Name	Lat.	Long.	Alt.	Temp.	Prec.	Source
Austria	Burgenland	128	Sopron	47.68	16.6	234	1871	1865	AlpImp; ZAMG
Austria	Carinthia	64	Klagenfurt-Airport	46.65	14.33	459	1813	1813	AlpImp; ZAMG
Austria	Lower Austria	152	Vienna-Hohe Warte	48.22	16.35	209	1775	1841	AlpImp; ZAMG
Austria	Salzburg	122	Salzburg-Airport	47.80	13.00	430	1842	1839	AlpImp; ZAMG
Austria	Styria	53	Graz-University	47.08	15.45	377	1837	1837	AlpImp; ZAMG
Austria	Tyrol	59	Innsbruck-University	47.27	11.38	609	1777	1858	AlpImp; ZAMG
Austria	Upper Austria	75	Linz	48.30	14.28	263	1816	1852	AlpImp; ZAMG
Austria	Vienna	152	Vienna-Hohe Warte	48.22	16.35	209	1775	1841	AlpImp; ZAMG
Austria	Vorarlberg	42	Feldkirch	47.27	9.62	440	1875	1876	AlpImp; ZAMG
Belgium	Flanders	64280	Munte	50.93	3.37	55	1973	1973	TUT
Belgium	Wallonia	64560	Florennes	50.23	4.65	279	1973	1973	TUT
Croatia	-	153	Zagreb-Gric; Split	45.82	15.98	162	1861	1862	AlpImp; TUT
Czech Republic	-	165	Brno-Turany	49.16	16.7	241	1848	1805	AlpImp; TUT
France	Alsace	135	Strasbourg-Entzheim-Airport	48.55	7.64	150	1973	1973	AlpImp; TUT
France	Aquitaine	75240	Agen	44.18	0.60	61	1973	1973	TUT
France	Auvergne	74600	Clement-Ferrand	45.78	3.16	332	1973	1973	TUT
France	Brittany	71300	Rennes	48.06	-1.73	37	1973	1973	TUT
France	Burgundy	72800	Dijon	47.26	5.08	222	1973	1973	TUT
France	Centre	72490	Orleans	47.98	1.75	126	1973	1973	TUT
France	Champagne-Ardenne	70700	Reims	49.30	4.03	95	1973	1973	TUT
France	Corsica	77610	Ajaccio	41.91	8.80	6	1973	1973	TUT
France	Franche-Comté	72880	Besancon	47.25	5.98	307	1973	1973	TUT
France	Île-de-France	71490	Paris-Orly	48.71	2.40	89	1973	1973	TUT
France	Languedoc-Roussillon	76350	Carcassonne	43.21	2.31	130	1973	1973	TUT
France	Limousin	74340	Limoges	45.86	1.18	396	1973	1973	TUT
France	Lorraine	93	Nancy-Essey-Tomblaine-Airport	46.00	8.97	273	1973	1973	AlpImp; TUT
France	Lower Normandy	70270	Caen	49.18	-0.45	78	1973	1973	TUT
France	Midi-Pyrénées	76300	Toulouse-Blagnac	43.63	1.36	152	1973	1973	TUT
France	Nord-Pas-de-Calais	70150	Lille	50.56	3.10	48	1973	1973	TUT

S2 Table (continued). Overview over the climatic data.

Country	Region	No.	Name	Lat.	Long.	Alt.	Temp.	Prec.	Source
France	Pays de la Loire	72300	Angers	47.48	-0.56	57	1973	1973	TUT
France	Picardy	70550	Beauvais-Lille	49.46	2.11	109	1973	1973	TUT
France	Poitou-Charentes	74120	Cognac	45.66	-0.31	30	1973	1973	TUT
France	Provence-Alpes-Côte d'Azur	75880	Saint-Auban-sur-Durance	44.06	6.00	460	1973	1973	TUT
France	Rhône-Alpes	74860	Grenoble	45.36	5.33	384	1973	1973	TUT
France	Upper Normandy	70370	Rouen	49.38	1.18	157	1973	1973	TUT
Germany	Baden-Württemberg	136	Stuttgart	48.83	9.20	311	1792	1807	AlpImp; DWD
Germany	Bavaria	193	Regensburg	49.03	12.10	366	1773	1800	AlpImp; DWD
Germany	Brandenburg & Berlin	403	Berlin-Dahlem	52.46	13.30	51	1876	1848	DWD
Germany	Hesse	1420	Frankfurt am Main	50.05	8.60	112	1757	1826	DWD
Germany	Lower Saxony & Bremen	2014	Hannover	52.47	9.68	55	1856	1856	DWD
Germany	Mecklenburg-Vorpommern	4271	Rostock	54.18	12.08	4	1947	1947	DWD
Germany	North Rhine-Westphalia	3	Aachen	50.78	6.09	202	1891	1891	DWD
Germany	Rhineland-Palatinate & Saarland	5100	Trier	49.75	6.66	265	-	1806	DWD
Germany	Saxony	1048	Dresden	51.13	13.76	227	1960	1960	DWD
Germany	Saxony-Anhalt	2932	Halle	51.44	12.24	131	1972	1972	DWD
Germany	Schleswig-Holstein & Hamburg	4466	Schleswig	54.53	9.55	43	1947	1947	DWD
Germany	Thuringia	1270	Erfurt	50.98	10.96	316	1951	1951	DWD
Hungary	Central Hungary	215	Budapest-Lörinc-Airport	47.45	19.22	130	1780	1841	AlpImp; TUT
Hungary	historical Hungary	215	Budapest-Lörinc-Airport	47.45	19.22	130	1780	1841	AlpImp
Hungary	Northern Hungary	127720	Miskolc	48.10	20.76	232	1973	1973	TUT
Hungary	Northern Transdanubia	137	Szombathely	47.27	16.63	221	1874	1865	AlpImp; TUT
Hungary	Southern Transdanubia	104	Pécs	46.06	18.17	150	1871	1854	AlpImp; TUT
Hungary	Trans-Tiszanian Region	128820	Debrecen	47.48	21.60	108	1973	1973	TUT
Italy	Tuscany	6	Arezzo	43.45	11.88	274	1973	-	AlpImp

S2 Table (continued). Overview over the climatic data.

Country	Region	No.	Name	Lat.	Long.	Alt.	Temp.	Prec.	Source
Luxemburg	-	65900	Luxembourg	49.61	6.21	376	1949	1949	TUT
Netherlands	Veluwe	62400	Amsterdam_Airport_Schiphol	52.30	4.76	-4	1973	1973	TUT
Poland	Central Poland	123750	Warszawa-Okecie	52.16	20.96	106	1973	1973	TUT
Poland	Eastern Poland	125850	Sandomierz	50.70	21.71	218	1989	1989	TUT
Poland	Northern Poland	122720	Olsztyn	53.76	20.41	137	1973	1973	TUT
Poland	Northwestern Poland	123300	Poznan	52.41	16.38	86	1973	1973	TUT
Poland	Podlaskie	122950	Bialystok	53.10	23.16	151	1973	1973	TUT
Poland	Southern Poland	125660	Krakow	50.08	19.8	237	1973	1973	TUT
Poland	Southwestern Poland	124240	Wroclaw_Ii	51.10	16.88	120	1973	1973	TUT
Slovakia	-	57	Hurbanovo	47.87	18.20	124	1872	1871	AlpImp; TUT
Spain	Villuercas	82610	Cáceres	39.46	-6.33	405	1973	-	TUT
Switzerland	Central Switzerland	4	Altdorf	46.87	8.63	449	1864	1864	AlpImp; TUT
Switzerland	Eastern Switzerland	50	Glarus	47.04	9.07	515	1864	1872	AlpImp; TUT
Switzerland	Espace Mittelland	17	Bern-Liebefeld	46.93	7.42	565	1777	1856	AlpImp; TUT
Switzerland	Lake Geneva region	126	Sion	46.23	7.37	482	1864	1861	AlpImp; TUT
Switzerland	Northwestern Switzerland & Zurich	157	Zürich-Meteo-Schweiz	47.38	8.57	556	1830	1830	AlpImp; TUT
Switzerland	Ticino	80	Lugano	46.00	8.97	273	1864	1861	AlpImp; TUT

The table gives the number of the climate station used (*no.*; corresponding to the respective source) and its name (*name*) for each region as well as the latitude (*lat.*), longitude (*long.*), and altitude (*alt.*) of the station. Further, the year of the oldest data point for monthly mean temperature (*temp.*) and monthly precipitation sum (*prec.*) is given. In the last column the *source* of the data is shown. The climatic data were acquired from the AlpImp-Project [1], the Central Institute for Meteorology and Geodynamics (ZAMG, www.zamg.ac.at), the German weather service (DWD; www.dwd.de), and from www.tutiempo.net (TUT). Data from the AlpImp-Project [1] were homogenized and only available until 2006 so that a second source was used for newer data. For incomplete monthly data points from TUT the mean temperature was calculated anyway and monthly precipitation sum was extrapolated to the full month if there were at least 20 days of data available in the respective month.

References

1. Auer I, Böhm R, Jurkovic A, Lipa W, Orlik A, Potzmann R, et al. HISTALP—historical instrumental climatological surface time series of the Greater Alpine Region. Int J Climatol. 2007;27(1):17-46. doi: 10.1002/joc.1377.
