# Supplementary material for: What Is a Mild Winter? Regional Differences in Within-Species Responses to Climate Change
Source: PLoS One. 2015 Jul 9;10(7):e0132178. doi: 10.1371/journal.pone.0132178 (PMC4497731; doi:10.1371/journal.pone.0132178)
Supplement: S3 Table — These results have been obtained using the zero method and are given in comparison to Table 1 showing the results obtained with the natural-average method. (PDF) [file pone.0132178.s006.pdf]

S3 Table. Model-averaged parameters calculated with the zero-method.

(a) European data set (*n* = 2075)

term	estimate	SE
**winter temperature**	0.188	0.025
**long-term winter temperature**	-0.263	0.033
**prior autumn temperature**	0.179	0.027
**population density**	-0.065	0.016
**summer temperature**	-0.041	0.022
**winter temperature : long-term winter temperature**	-0.047	0.026
prior spring temperature	0.024	0.022
prior summer temperature	-0.011	0.019
long-term summer temperature	-0.005	0.019
long-term summer precipitation	0.008	0.017
winter temperature : population density	0.011	0.023
long-term yearly precipitation	0.004	0.015
spring temperature	-0.003	0.014
summer temperature : long-term summer temperature	-0.002	0.011

(b) Austrian data set (*n* = 135)

term	estimate	SE
**winter temperature**	0.237	0.064
**mast**	0.284	0.112
**mast : winter temperature**	-0.910	0.245
**prior autumn temperature**	0.162	0.061
**long-term winter temperature**	-0.141	0.081
population density	-0.065	0.092
area under cultivation of potatoes	-0.043	0.059
area under cultivation of corn	-0.035	0.065
summer temperature	-0.022	0.043
prior spring temperature	-0.005	0.029
winter temperature : long-term winter temperature	-0.009	0.050
mast : long-term winter temperature	-0.098	0.294
mast : population density	-0.012	0.108

Parameters estimate and standard error (SE) for each term included in the multi-model averages of the European data set (a) and the Austrian subset (b), calculated with the zero-method [1, 2]. Interaction terms are indicated by colons, significant predictor variables are highlighted bold. See methods for the definition of seasons.

References

1. Nakagawa S, Freckleton RP. Model averaging, missing data and multiple imputation: a case study for behavioural ecology. Behav Ecol Sociobiol. 2011;65(1):103-16. doi: 10.1007/s00265-010-1044-7.

2. Grueber CE, Nakagawa S, Laws RJ, Jamieson IG. Multimodel inference in ecology and evolution: challenges and solutions. J Evol Biol. 2011;24(4):699-711. doi: 10.1111/j.1420-9101.2010.02210.x.
