# Supplementary material for: What Is a Mild Winter? Regional Differences in Within-Species Responses to Climate Change
Source: PLoS One. 2015 Jul 9;10(7):e0132178. doi: 10.1371/journal.pone.0132178 (PMC4497731; doi:10.1371/journal.pone.0132178)
Supplement: S1 File — (PDF) [file pone.0132178.s007.pdf]

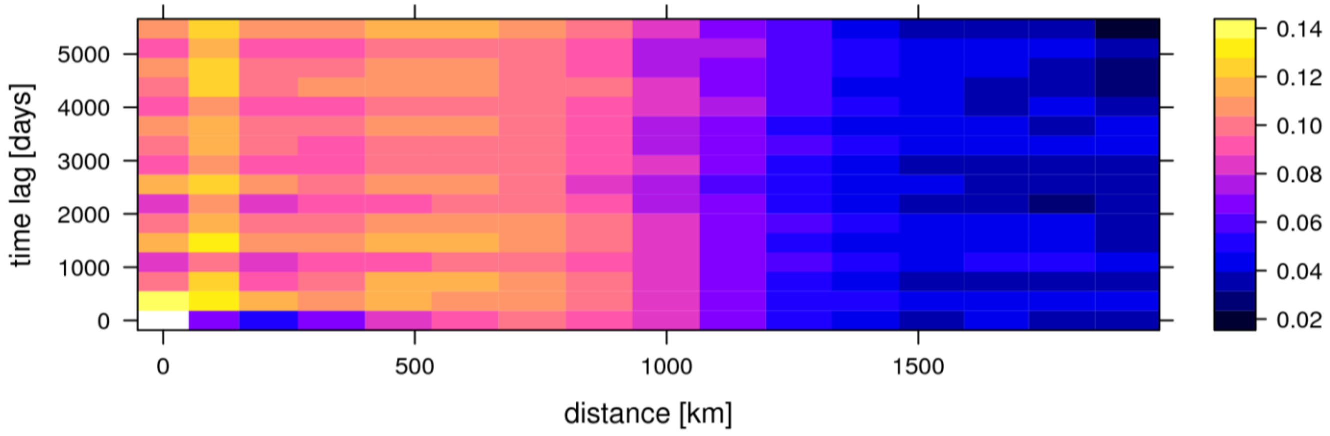

Figure A. Spatio-temporal semivariogram of the residuals of the European multi-model average. Colour codes refer to γ-values indicated by the legend.

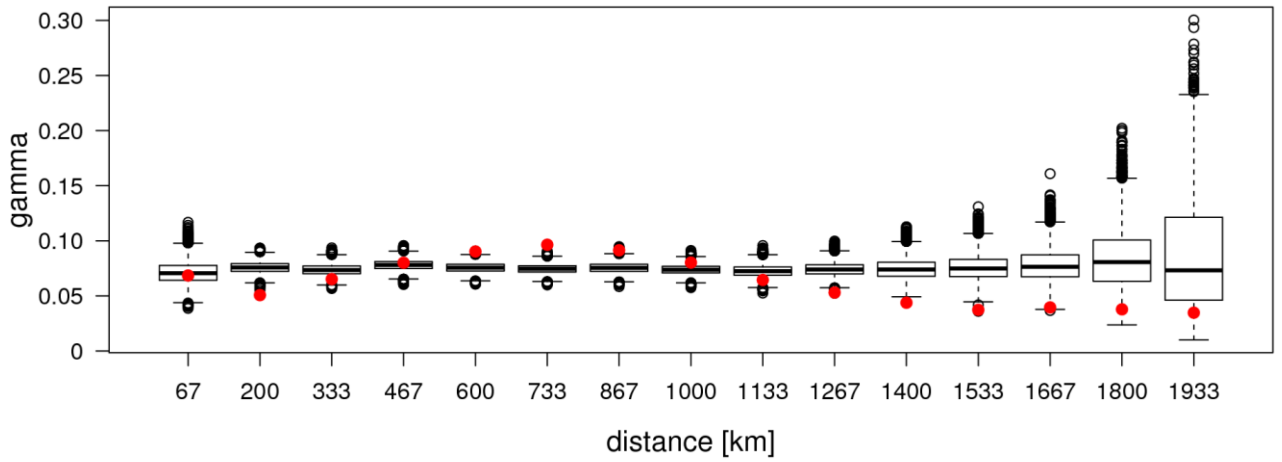

Figure B. Bootstrapped spatial autocorrelation structure of the residuals of the European multi-model average. Bootstrapping was performed by randomly sampling 75% of the residuals and calculating the respective spatial autocorrelation structure without any time lag for 10,000 times. Red points show the original semivariogram including the full data set.
